# New insights into ANGPLT3 in controlling lipoprotein metabolism and risk of cardiovascular diseases

**DOI:** 10.1186/s12944-018-0659-y

**Published:** 2018-01-15

**Authors:** Xin Su, Dao-quan Peng

**Affiliations:** Department of Cardiovascular Medicine, the Second Xiangya Hospital, Central South University, Changsha, Hunan 410011 China

**Keywords:** ANGPTL3, Lipoprotein metabolism, Lipoprotein lipase, Dyslipidemia, Cardiovascular diseases

## Abstract

Dyslipidemia, characterized by elevation of plasma low density lipoprotein cholesterol (LDL-C), triglyceride (TG) and reduction of plasma high density lipoprotein cholesterol (HDL-C), has been verified as a causal risk factor for cardiovascular diseases (CVD), leading to a high mortality rate in general population. It is important to understand the molecular metabolism underlying dyslipidemia in order to reduce the risk and to develop effective therapeutic approaches against CVD. ANGPTL3 (human) or Angptl3 (mouse), one member of the angiopoietin-like protein (ANGPTL) family, has been identified as an important regulator of lipid metabolism by inhibiting LPL and EL activity. Results have demonstrated that inactivation of Angptl3 in mice could obviously reduce the level of TG, LDL-C and the atherosclerotic lesion size, leading to a lower risk for dyslipidemia and CVD. Additionally, in humans, carriers with homozygous LOF mutations in ANGPTL3 have lower plasma LDL-C, TG levels and lower risk of atherosclerosis compared to the non-carriers. Here, we collect the latest data and results, giving a new insight into the important role of ANGPTL3 in controlling lipoprotein metabolism. Finally, we introduce two update reports on the antisense oligonucleotide and monoclonal antibody-based inactivation of ANGPTL3 in human clinical trials, to identify that ANGPTL3 could be a novel and effective target for the treatment of dyslipidemia and CVD.

## Background

Fats from our daily diet will be transported into circulation in the form of lipoprotein particles containing apolipoprotein, cholesterol, and triglycerides (TGs) [1, 2]. In humans, TGs are always packed after feeding both in the small intestine in chylomicrons (CM) and in the liver in very low density lipoproteins (VLDL) [3]. Lipoprotein lipase (LPL), a hydrolytic enzyme produced by many tissues, is rate-limiting for the removal of lipoprotein TGs from the circulation [4]. After deprivation of TGs by LPL, the remnants of CM [5] and VLDL [6] are cleared via specific hepatic receptors, while the remaining free fatty acids (FFA) are taken up by peripheral tissues as sources of energy [3]. This progress can keep lipid metabolism balance in humans. However, once this balance is broken, the individuals may suffer from dyslipidemia, characterized by elevation of plasma LDL-C, TG and reduction of plasma HDL-C [7, 8]. Evidence have revealed that dyslipidemia has a closely relationship with the morbidity of cardiovascular diseases (CVD) [9, 10], which can lead to a high mortality rate in general population in both developed and developing countries all over the world [11]. Therefore, in order to reduce the risk of CVD and to develop effective therapeutic approaches against these conditions, it is important to understand the molecular metabolism underlying dyslipidemia.

By genetic variants, numerous results have demonstrated that lots of disturbances in lipid metabolism have been detected in humans, such as reduced LPL activity [12], impaired LDL receptor activity [13] and disturbed lipoprotein secretion [14]. According to the type of disturbances, treatment of dyslipidemia involves dietary changes, exercise, lipid-lowering drugs, and even gene-targeting therapy. Currently, the existing pharmaceuticals which target on lowering LDL-C and TG level and on elevating HDL-C level, such as statins, fibrates, proprotein convertase subtilisin kexin 9 (PCSK9) inhibitors, cholesteryl ester transfer protein (CETP) inhibitors and fish oil, have been utilized alone or in various combinations to treat dyslipidemia [15–18]. However, those medicines are no panacea. For instance, the adverse effects of statins, such as statin-associated new onset diabetes and hepatic toxicity, may limit the use of statins [19]. Moreover, it is difficult to promote the gene-targeting therapy in developing countries currently due to the high cost and immature technology [20]. Taking into account these factors, the researchers have to search for additional targets to further reduce the risk of CVD.

Recently, a specific family of secretory proteins have been named ‘angiopoietin-like proteins’ (ANGPTLs), which shares a structural similarity to angiopoietins, the key factors that regulate angiogenesis [21]. The ANGPTLs contain eight members and all play an important role of plasma lipid metabolism in humans and animals [22]. Of these eight ANGPTLs, the effect of ANGPTL3 in controlling plasma lipid metabolism has sparked most interest of researchers in recent years. Actually, results of two large scale researches published in 2017 have manifested the inhibition ANGPTL3 by monoclonal antibody or antisense oligonucleotides can obviously influence the concentration of lipid and reduce the risk of dyslipidemia and CVD. Here, we review our current understanding of ANGPTL3, especially focusing on its role in the regulation of plasma lipid metabolism and its status as an effectively therapeutic target for dyslipidemia and CVD.

## The structural features and expression of ANGPTL3

Early in 2002, ANGPTL3 was identified by researching in KK mice and KK/Snk mice [23]. As is known to us, KK mice are a kind of animal models with obesity, hyperglycemia, hypercholesterolemia and hypertriglyceridaemia; conversely, KK/Snk mice, a sub-strain of the KK obese mice, have the hypolipidaemia with lower levels of TG, total cholesterol and FFA in plasma [23]. By analyzing the gene sequence in KK/Snk mice, the researchers found that KK/San mice carried a 4 bp insertion in exon 6 of Angptl3, causing a genetic frameshift mutation and the early termination of gene coding, which led to the decline in plasma lipid levels. Both injecting KK/Snk mice with recombinant Angptl3 protein and overexpressing Angptl3 gene could obviously raise plasma levels of TG, total cholesterol and FFA [23, 24]. Collectively, researches declared to established Angptl3 as the gene responsible for the lipid phenotype in KK/Snk mice.

Human ANGPTL3 gene is located on chromosome 1p31 while mice Angptl3 is located on chromosome 4 [25]. This gene began to express at the early stage of liver development physically [26]. The ANGPTL3 protein is a 460-amino-acid (aa) polypeptide with a distinctive signal peptide sequence, a N-terminal helical domain (predicted to form dimeric or trimeric coiled-coil structures) and a C-terminal globular fibrinogen homology domain. The N-terminal coiled-coil region (17-207aa) affects plasma TG levels via reversibly inhibiting catalytic activity of LPL while the fibrinogen-like domain (207-460aa) binds to integrin αvβ3 receptor and affects angiogenesis, which is similar to the function of angiopoietins [27]. A short linker region (at 221–222 and 224–225) between N- and C-terminal domains is a special zone, which has been verified to be split by furin. Existed results have shown that the truncated form of cleavage ANGPTL3 could reinforce the inhibitory activity of LPL and endothelial lipase (EL), suggesting that the cleavage type of ANGPTL3 may function more effectively [28, 29].

Being a kind of hepatokine, ANGPTL3 is exclusively expressed in the liver and is secreted into circulation where it goes through cleavage by hepatic proprotein convertases [25]. The expression of ANGPTL3 is affected by many factors. Studies have verified that ANGPTL3 is a direct target of LXR and that T0901317, a synthetic LXR-selective agonist, could upregulate the gene expression of Angptl3 in mice liver, resulting in corresponding change in concentration of lipid in plasma [22, 30, 31]. Meanwhile, treatment with T0901317 led to a dose-dependent increase of ANGPTL3 mRNA in HepG2 cells [32]. These data revealed that LXR agonists could induce the expression of Angptl3. In addition, studies have found that some factors, like PPARγ, statins and leptin, could obviously suppress mRNA expression of Angptl3 in mice, and both insulin and rosiglitazone could reduce ANGPTL3 secretion by a dose-dependent manner in immortalized human hepatocytes [33]. Another remarkable point to note is that even though the expression of Angptl3 mRNA in mice could not be regulated by fasting or feeding, the effects of ANGPTL3 on inhibiting LPL are most pronounced in the feeding state [34]. The mechanism seems to be referred to the interaction between ANGPTL3 and ANGPTL8, since ANGPTL8 could be induced in the fed or refed state [35].

## The population studies of ANGPTL3 gene variants in humans

There were numerous studies focusing on genetic variants of ANGPTL3 in humans in the past decade; and the results emphasized the significance genetic variants of ANGPTL3 in controlling lipid metabolism in humans. Three SNPs at loci near ANGPTL3, including rs1748195, rs12130333 and rs2131925, have been confirmed to associate with plasma lipid concentrations by genome wide association studies (GWAS). Besides these three SNPs loci, rs12130333 was closely associated with the Fredrickson hyperlipoproteinemia type 5, characterized by a mixed hyperlipidaemia of elevated levels of VLDL and CM [36]. On the contrary, rs2131925 was shown to be associated with a mean reduction in plasma TG of 4.94 mg/dl [37], and the patients carried rs12042319 was also presented lower LDL-C and TG levels [38]. Another exon sequencing result of the 7 exons of a multi-ethnic sample of 3551 individuals in the Dallas Heart Study provided evidence for a link between the ANGPTL3 gene variant and plasma TG levels. A total of 35 non-synonymous sequence variants, including non-sense mutations and frameshift mutation, were identified. All the individuals carried LOF mutations in ANGPTL3 presented the lowest level of TG levels [31]. Furthermore, a type of gene variant called M259 T in ANGPTL3 was also reported to associate with lower plasma TG levels among African Americans in the Atherosclerosis Risk in Communities study [31]. Since this kind of variant is mainly present in African Americans, it needs further studies to observe whether M259 T is associated with lower plasma TG levels in other ethnic group.

Moreover, another exome sequencing of 15,994 genes of two individuals from a family with hypobetalipoproteinaemia (very low plasma level of LDL-C, HDL-C and TG) led to an identification of two novel ANGPTL3 gene variants, which gives the causal relationship between ANGPTL3 and familiar combined hypolipidaemia [39]. The two individuals were confirmed to carry compound heterozygotes with the nonsense mutations S17X and E129X in ANGPTL3, and these two carriers have the extremely low plasma LDL-C and TG level and almost no detectable ANGPTL3 in their plasma. Besides, the S17X carriers also have a higher post-heparin LPL activity and mass [31]. Of interest, the S17X was subsequently verified to cause familial hypobetalipoproteinaemia in an Italian family. The three generations carried S17X heterozygotes had significantly lower plasma LDL-C and TG levels compared to 21 non-mutation family members [40]. The latest report published in 2017 by Dewey and found the clearly relationship between the ANGPTL3 gene variant and plasma lipid levels, especially on LDL-C and TG levels [41].

## The function of ANGPTL3

### The role of ANGPTL3 on lipid metabolism by interfering lipoprotein clearance

The circulating level of TG is related with the lipolysis rate of TG-rich lipoprotein (TRLs). Three lipoprotein lipase, including LPL, EL, and hepatic lipase (HL), are responsible for the lipolysis of TG in lipoprotein. As mentioned above, LPL is the rate-limiting enzyme which plays a critical role in lipolysis of TRLs in the circulation [4] and the activity of LPL in white adipose tissue (WAT) is increased in feeding state while decreased in fasting state [42]. EL, located in the lumen of vascular endothelial cells, is more specific in hydrolysis of lipoprotein phospholipids, especially in HDL particles, rather than TG [43]. Previous results have demonstrated that both LPL and EL activity were increased in Angpt3−/− mice, accompanied with the accumulation of TRLs in BAT and muscle instead of WAT. These results suggested that and TG in apolipoprotein was imported into BAT and muscle instead of WAT, giving evidences that ANGPTL3 could obviously inhibit LPL [32] and EL activity [43], prevent TG from hydrolysis [44] and then accelerate the removal of particles rich in TG [45]. There was no report of enhanced HL activity in ANGPTL3 deficiency.

A question about the mechanism between ANGPTL3 deficiency and low LDL-C levels was put forward several years ago. Since LPL or EL does not have the function to hydrolyze cholesteryl esters, the question was still remains unsolved. In 2015, Wang observed that both wild-type mice and Ldlr, Lrp1 or ApoE knockout mice had almost equal levels of LDL-C after treating with Angptl3 antibody; in addition, every group had much at one extent of reduction in LDL-C levels [46]. Thus, we could infer from these results that the reduction of LDL-C in Angptl3 deficiency mice was not caused by increased cholesterol clearance through the LDL receptor. The decreased synthesis of VLDL-C may be the plausible explanation.

### The role of ANGPTL3 in influencing lipoprotein production

Several approaches can promote the hepatic VLDL synthesis, and the main approach relies on the validity of TG synthesized from the supply of plasma FFA from adipocytes lipolysis [47], VLDL and CM remnants [48] and the monosaccharide transported through the portal vein [29]. Meanwhile, the insulin signaling pathway can also influence hepatic VLDL synthesis and secretion because of the direct effect of insulin in decreasing the lipidation of TG-rich VLDL particles [49].

The scientists has already found that hepatic VLDL-TG secretion was no changed in KK/Snk mice compared to the wild-type KK mice, meanwhile, these KK/Snk mice also showed no significant difference in the production rates of apolipoprotein B-100 (apoB100) and the amount of secreted VLDL particles [50, 51]. Interestingly, studies have done to investigate the effect of ANGPTL3 on the lipolysis in 3 T3-L1 adipocytes and found that incubation with human ANGPTL3 could enhance the release of FFA into medium, similar to the effect of epinephrine [52]. These observations were in accordance with the reported low FFA levels in ANGPTL3 LOF mutation individuals. As KK/Snk mice exhibited extremely lower TG concentration and FFA in plasma, suggesting that reduced lipidation of VLDL in ANGPTL3-deficiency mice may be caused by decreased supply of FFA from the circulation into the liver, resulting a declined amount of TG in each VLDL particle. Additionally, another study using ANGPTL3 deficiency hepatocytes has found that silencing of ANGPTL3 could cause a shift in taking advantage of monosaccharide in plasma instead of FFA [53], which also provided the evidence to the theory of diluted FFA supplied into the liver.

Cholesterol and other cholesterol metabolites, either synthesized by the liver or ingested from our daily diet [54], are the natural ligands for LXR [55]. As described previously, the activated LXR could stimulate bile acid secretion and the progress of lipogenesis, meanwhile, it could promote reverse cholesterol transport mediated by HDL, thus protecting the liver from superabundant cholesterol [56]. Studies have shown that synthetic LXR ligands and a high cholesterol diet could induce the gene expression of hepatic ANGPTL3 [57]. As ANGPTL3 is a downstream target for LXR, it is worth noting that reduced availability of substrates may decrease hepatic synthesis of cholesterol, resulting in the secretion of cholesterol-poor VLDL in ANGPTL3-deficiency mice [58]. On the contrary, low LXR, and low ANGPTL3 expression (in ANGPTL3-deficiency or inactivation of ANGPTL3) may therefore be linked to a lower level of cholesterol in the liver [59]. Thus, we could infer from these results that the reduction of LDL-C in Angptl3 deficiency mice was caused, at least partly, by the decreased synthesis of VLDL-C. Trials are underway to further explore the potential of mechanism.

### The indirect effect of ANGPTL3 on lipid metabolism by interfering insulin sensitivity

Although the role of ANGPTL3 in lipid metabolism is relatively clear, the relationship between ANGPTL3 and insulin sensitivity remains uncertain. Only a few of studies demonstrated the role of ANGPTL3 in regulation of insulin sensitivity and glucose metabolism. In 2013, Rebciuc and colleagues compared insulin sensitivity in carriers of the homozygous and heterozygous LOF mutation in ANGPTL3 with non-carriers. They found that the plasma insulin, glucose, and homeostatic model assessment of insulin resistance (HOMA-IR) were significantly lower in homozygous subjects compared with heterozygotes and non-carriers subjects, suggesting that homozygotes had higher insulin sensitivity than that in heterozygotes and non-carriers subjects [40]. These data manifested ANGPTL3 may affect insulin sensitivity and play a role in modulating glucose metabolism in humans. Additionally, Wang et al. found that plasma concentrations of glucose and insulin are comparable between Angptl3−/− and wild-type genotypes in mice, and Angptl3−/− mice exhibited a higher uptake rate of the deoxyglucose in WAT, BAT and the heart [46]. In vitro studies, they used immortalized human hepatocytes and also found that ANGPTL3 deficiency can obviously increase deoxyglucose uptake [46]. Interestingly, the effect of ANGPTL3 on insulin and glucose metabolism was reciprocally. Data from Tikka et al. have revealed that both insulin and rosiglitazone could decrease the secretion of ANGPTL3 in a dose-dependent manner, and silencing of ANGPTL3 improved glucose uptake in hepatocytes by about 45% [29, 53].

Based on the fact of interaction of ANGPTL3 and insulin, we could propose the indirect effect of ANGPTL3 on lipid metabolism by interfering insulin sensitivity. Gathered with these conclusions, we can speculate that silencing of ANGPTL3 may improve insulin sensitivity and increase glucose uptake and utilize by peripheral organs.

### The function and mechanism of ANGPTL3 in inhibiting LPL activity

Since the function of ANGPTL3 in inhibiting LPL catalytic activity has been well demonstrated, researches have investigated the mechanism of ANGPTL3 and LPL activity. Firstly, the unique structure of ANGPTL3 peptide makes contribution to the inhibitory effect of ANGPTL3. The N-terminal coiled-coil region of ANGPTL3 peptide exists three important amino acids, aspartic acid, histidine and glutamine [60], which can prevent LPL from binding to glycosylphsphatidylinositol- anchored high-density lipoprotein binding protein 1 (GPIHBP1) [61], a protein expressed on capillary endothelial cells and transports LPL to the site of the capillary lumen. This progress can reduce the activity of LPL in humans. Additionally, in 2009, Yau and his group found that ANGPTL3 could accelerate the unfolding progress of LPL, as promoting the dissociation of catalytically active LPL dimers into inactive LPL monomers, leading to an obviously reduction of LPL activity [62]. They also found that though ANGPTL3 and ANGPTL4 had the similar but not identical pathways in reducing LPL activity, the efficiency of ANGPTL3 in inhibiting LPL is much weaker than that of ANGPTL4 [62]. Furthermore, Liu and his colleague demonstrated in 2010 for another potential mechanism of ANGPTL3 and LPL activity. They showed that ANGPTL3 stimulates the extracellular cleavage of LPL by furin, and the simulation could result in the dissociation of LPL from the cell surface and the reduction of the catalytic functions of LPL [32].

Thus, by synthesizing all the data mentioned above, we can speculate ANGPTL3 has a much lower effect on LPL than ANGPTL4. The mechanism of ANGPTL3 in LPL activity may involve unfolding dimer structure of LPL and preventing LPL from binding to GPIHBP1. Finally, an important difference between the two proteins is that ANGPTL3 inhibits LPL through an endocrine pathway, and the effect of ANGPTL4 on LPL may be through autocrine pathways and local paracrine.

### The function of ANGPTL3 in promoting dyslipidemia and the risk of CVD

There is considerable evidence revealing that elevated level of plasma LDL-C is the independent risk factors for CVD [16, 63, 64]. Recently, other studies have also shown that elevated plasma TG levels also play an important role in the development of CVD [65, 66]. Targeting on LDL-C and TG may effectively prevent the progress of CVD. As both individuals with LOF mutations in ANGPTL3 or mice with Angptl3 deficiency have lipid-lowering lipoprotein profile and reduced plasma level of LDL-C and TG, we could speculate that ANGPTL3 deficiency may lead a low risk of CVD in humans.

The report from Deway revealed that in 45,226 individuals, carriers of a LOF mutations in ANGPTL3 had 27% lower TG levels, and 9% lower LDL-C levels than non-carriers [41]. In 13,102 individuals with CVD, the presence of a LOF mutations in ANGPTL3 was associated with a 41% lower risk of CVD. Secondly, the authors used APOE*3 Leiden.CETP mice to investigate the effects of a monoclonal antibody against ANGPTL3 antibody, Evinacumab. As the author reported, Evinacumab treatment was associated with an obviously reduction in total cholesterol level and TG level [41]. Moreover, Evinacumab could also led to a significantly greater decreased in atherosclerotic plaque area in the aortic root [41]. These results are a powerful illustration of the relationship between ANGPTL3 and the risk of CVD.

Another latest report is from Stitziel and his colleagues published in 2017, which confirmed the direct relationship among ANGPTL3, dyslipidemia and the risk of CVD through three different methods. Firstly, Stitziel found that the three individuals, carrying homozygous mutations in ANGPTL3, showed no evidence of coronary atherosclerotic plaque compared with non-carriers [67]. Secondly, they used a meta-analysis to investigate more than 180,000 individuals, including 21,980 individuals with CVD, and observed that approximately 1 in 309 people was a heterozygous carrier for an LOF mutation by ANGPTL3 gene sequencing. Even though this mutation is rare, the heterozygous carriers had about 34% lower risk of CAD compared with non-carriers. It is notable that a 17.3% reduction in plasma TG and a 11.8% reduction in LDL-C. However, the plasma HDL-C also reduced about 5.2%, although the differences are not statistically significant [67]. This part of data revealed that LOF mutation in ANGPTL3 could reduce the risk of dyslipidemia in humans probably via reducing the level of LDL-C and TG, not the levels of HDL-C [39, 67]. Finally, the research group observed the risk of myocardial infarction was reduced by 29% compared with the highest tertile of individuals with the lowest tertile of ANGPTL3 in 1493 patients with CVD and 3231 controls, after adjustment for plasma LDL cholesterol and TG. By far, this was the only one result revealing the relationship between plasma concentration of ANGPTL3 and the risk of CAD [67], suggesting that ANGPTL3 might influence the progress of CAD via an independent method beyond its function in controlling lipid metabolism.

Besides the inhibitory property to LPL, ANGPTL3 can also suppress the activity of EL [23, 68], which could explain the phenomenon for the decreased HDL-C level in mice with Angptl3 deficiency and in individuals carrying LOF mutation in ANGPTL3. However, data from Stitziel published in 2017 showed that in 20,092 individuals, the LOF mutation in ANGPTL3 carriers exhibited 5.2% reduction in HDL-C [67]. Meanwhile, Deway also presented a result that in 45,036 participants in DiscovEHR study, the level of HDL-C reduced about 6.1% in LOF mutation of ANGPTL3 carriers [41]. Thus, the biochemical mechanism and relationship between ANGPTL3 and EL remains elusive.

### The cooperation between ANGPTL3 and ANGPTL8 in controlling lipid metabolism

Both ANGPTL3 and ANGPTL8 are secreted proteins and show a effect of inhibiting LPL-mediated plasma TG hydrolysis. Co-expression with ANGPTL3 can greatly enhance the secretion of ANGPTL8 [69]. Several researches have investigated whether these two ANGPTL proteins could interact for the regulation of LPL activity. They found ANGPTL3 and ANGPTL8 could cooperate in the regulation of plasma TG levels [70]. As mentioned previously, Angptl3 and Angptl8 overexpression respectively could both led to dramatically increased serum TG levels in mice, however, Quagliarini found that in Angptl3−/− mice, Angptl8 seems to be inactive while in the presence of Angptl3, Angptl8 can reduce the activity of LPL more effectively. On the contrary, in the absence of ANGPTL8, the ability of ANGPTL3 to raise plasma TG levels decreased mildly [37]. In another study, Chi also observed that ANGPTL3 or ANGPTL8 alone could only inhibit LPL at concentrations that far exceeded physiological levels, especially when LPL was bound to its endothelial cell receptor GPIHBP1. However, ANGPTL8 could promote the ability of ANGPTL3 to inhibit LPL, but only when the two proteins were co-expressed in the same 293 T cell [70]. Protein interaction assays in this study also revealed that ANGPTL8 greatly increased the ability of ANGPTL3 to bind LPL, indicating that a physical interaction between ANGPTL3 and ANGPTL8 might cause this functional cooperation.

Indeed, biochemical studies have shown that ANGPTL3 and ANGPTL8 could be co-immunoprecipitated in the plasma of mice or in the culture medium of cells expressing both proteins [71, 72]. Intensive investigation has been did by Haller and some evidence have been provided to verify whether ANGPTL3 needs to form a complex with ANGPTL8 to make itself more active in inhibiting LPL. Using a mutated form of ANGPTL3 that lacks LPL inhibitory activity, Haller demonstrated that ANGPTL3 activity was not required for its ability to activate ANGPTL8 [73], and co-expression of Angptl3 and Angptl8 could lead a far more efficacious increased in TG in mice than Angptl3 alone [73, 74], suggesting the major inhibitory activity of this complex derives from ANGPTL8. An antibody to the C-terminus of ANGPTL8 could reverse inhibitory effect to LPL of ANGPTL8 in the presence of ANGPTL3. The antibody did not disrupt the ANGPTL8-ANGPTL3 complex but comes in close proximity to the LPL inhibitory motif in the N-terminus of ANGPTL8 [73]. Collectively, these data implied that ANGPTL3 could serve as a binding partner and activator of ANGPTL8 and ANGPTL8 could promote its own ability to inhibit LPL and increases plasma TG levels in the presence of ANGPTL3.

## Could ANGPTL3 be the next PCSK9 and a target lipid-lowering therapy?

As is known to us, proprotein convertase subtilisin/kexin type 9 (PCSK9) plays an important role in human lipid metabolism [75]. In ACC 2017, a large-scale clinical study called *Further cardiovascular OUtcomes Research with PCSK9 Inhibition in subjects with Elevated Risk* (FOURIER) trial was published, which focused on the effect of PCSK9 inhibitor to atherosclerosis. The results showed that in the Evolocumab (a kind of PCSK9 inhibitor) treatment group, the level of LDL-C was reduced by 59%, reaching an average of 30 mg/dl; additionally, a 15% reduction in the primary end point, a 20% reduction in the primary secondary endpoint, and no adverse events increased in the Evolocumab treatment group [76]. This study demonstrated that PCSK9 inhibitors could obviously reduce the risk of CVD by down-regulating LDL-C concentration. Moreover, in the ESC2017, the subgroup analysis of FOURIER study showed that the primary cardiovascular end point was lower linearly with the decreased of LDL-C level. Even when LDL-C is less than 0.26 mmol/L, there was a decrease in the primary end point [77, 78].

Consistent with PCSK9, the significant effect of ANGPTL3 on plasma lipid levels has raised interest in ANGPTL3 as a therapeutic target for the treatment of dyslipidemia and CVD. Besides the results from Deway mentioned above, another report from a phase I randomized, placebo-controlled clinical trial by Graham’s group gave evidence about the antisense oligonucleotide against the gene of ANGPTL3 in healthy volunteers. Participants who received the therapy by 60 mg/week and presented a mean reduction in TG levels of 50%, in LDL-C levels of 33%, and in HDL-C levels of 27% [79]. In these two studies, researchers observed that participants displayed the expected reduction in plasma levels of TG or LDL-C; and the progression of atherosclerosis in mice also retarded by using Evinacumab. It seems that both antisense oligonucleotide against ANGPTL3 gene and monoclonal antibody of ANGPTL3 have the effect in reducing the risk of CVD. These observations strongly suggested that molecular therapy for ANGPTL3 in reducing the incidence of cardiovascular events is safe and effective. The results were similar to those previously described with respect to PCSK9 inhibitors [80]. However, the two large scale studies had some limitations in the followed aspects. Firstly, the number of participants in clinical trials was limited, and the main subjects of DiscovEHR researched by Deway were European people, so it remains unclear of the function of ANGPTL3 in lipoprotein metabolism in Asian or other population. Secondly, these large studies used the animal models to investigate the process of atherosclerosis, but the effect of ANGPTL3 in preventing the process of atherosclerosis in humans remains unclear. Moreover, during the studies, some participants suffered from headache and mild hepatic injury after injected Evinacumab. Seeing these limitations, we still have a wider space of research on the role of ANGPTL3.

Overall, the results of human clinical trials for ANGPTL3 are sufficient to be satisfactory. The ongoing phase III clinical trial is reasonable and the results are worth waiting. Since dyslipidemia could increase the risk of CVD, drugs targeting ANGPTL3 may have considerable hope for reducing the risk of dyslipidemia, obesity and CVD.

## Conclusions

Since first discovered in 2002, ANGPTL3 has been considered as an important and novel regulator of plasma lipid. Numerous studies have already confirmed ANGPTL3 to be a promising target in the pharmacological therapy of dyslipidemia and CVD. However, lots of questions about the role of ANGPTL3 in lipid metabolism are still remaining. For instance, as mentioned above, injecting the ANGPTL3 adenovirus, which aims to overexpress ANGPTL3, could result in an increase in plasma levels of TG and FFA. The upregulation of plasma TG levels was caused by the inhibitory effect of ANGPTL3 to LPL, and the increased FFA was resulted from the enhanced lipolysis of adipocytes [24, 52]. Thus, the question is how ANGPTL3 affects adipose tissue lipolysis. In particular, we need a better understanding of the molecular mechanisms involved in mediating the effect of ANGPTL3 on adipose tissue lipolysis. As is known to us, hormone sensitive lipase (HSL), perilipin, and aquaporin adipose (AQPap) are the proteins involved in the lipolysis of adipose cells. Interestingly, studies have shown that although ANGPTL3 could strongly bind on adipose tissue, the mRNA expression level of those three lipolytic genes were not altered in KK/Snk mice, compared to KK mice [52]. It seems that ANGPTL3 does not affect adipocyte lipolysis through influencing the lipolytic genes, nevertheless, as the functions of those proteins are mainly regulated post-translationally, the effects of ANGPTL3 on the modifications of these proteins also need to be investigated.

Collectively, data have shown that ANGPTL3 is an important regulator of LPL activity and plasma TG clearance. Moreover, besides inhibiting LPL activity in all tissues, ANGPTL3 could also promote the uptake of plasma TG in WAT by preferentially suppressing the clearance of TRLs in oxidative tissues such as the heart and BAT. We can also observe a significant hypolipidemic phenotype in the carriers of ANGPTL3 LOF mutations, suggesting that ANGPTL3 LOF mutations can be served as a target for hyperlipidemia. Therefore, we can use the relevant technology to inactivate ANGPTL3 to treat dyslipidemia, obesity and CVD. In conclusion, ANGPTL3 is an important hepatic derived regulator of plasma lipoprotein metabolism. Clinical trials are underway to further explore the potential of inactivating ANGPTL3 as a therapeutic target for dyslipidemia and CVD.

## Abbreviations

ANGPTL: Angiopoietin-like
apoB: Apolipoprotein B
apoE: Apolipoprotein E
BAT: Brown adipose tissue
CAD: Coronary artery disease
CETP: Cholesteryl ester transfer protein
CM: Chylomicron
CVD: Cardiovascular disease
EL: Endothelial lipase
FFA: Free fatty acids
FOURIER: *Further cardiovascular OUtcomes Research with PCSK9 Inhibition in subjects with Elevated Risk*
GPIHBP1: Glycosylphsphatidylinositol- anchored high-density lipoprotein binding protein 1
GWAS: Genome-wide association study
HL: Hepatic lipase
HOMA-IR: Homeostatic model assessment of insulin resistance
LDL: Low density lipoprotein
LOF: Loss-of-function
LPL: Lipoprotein lipase
LXR: Liver X receptor
PCSK9: Proprotein convertase subtilisin kexin 9
SNP: Single nucleotide polymorphism
TG: Triglyceride
VLDL: Very low density lipoprotein
WAT: White adipose tissue
